# Efficient algorithms for training the parameters of hidden Markov models using stochastic expectation maximization (EM) training and Viterbi training

**DOI:** 10.1186/1748-7188-5-38

**Published:** 2010-12-09

**Authors:** Tin Y Lam, Irmtraud M Meyer

**Affiliations:** 1 Centre for High-Throughput Biology, Department of Computer Science and Department of Medical Genetics, 2366 Main Mall, University of British Columbia, Vancouver V6T 1Z4, Canada

## Abstract

**Background:**

Hidden Markov models are widely employed by numerous bioinformatics programs used today. Applications range widely from comparative gene prediction to time-series analyses of micro-array data. The parameters of the underlying models need to be adjusted for specific data sets, for example the genome of a particular species, in order to maximize the prediction accuracy. Computationally efficient algorithms for parameter training are thus key to maximizing the usability of a wide range of bioinformatics applications.

**Results:**

We introduce two computationally efficient training algorithms, one for Viterbi training and one for stochastic expectation maximization (EM) training, which render the memory requirements independent of the sequence length. Unlike the existing algorithms for Viterbi and stochastic EM training which require a two-step procedure, our two new algorithms require only one step and scan the input sequence in only one direction. We also implement these two new algorithms and the already published linear-memory algorithm for EM training into the hidden Markov model compiler HMM-CONVERTER and examine their respective practical merits for three small example models.

**Conclusions:**

Bioinformatics applications employing hidden Markov models can use the two algorithms in order to make Viterbi training and stochastic EM training more computationally efficient. Using these algorithms, parameter training can thus be attempted for more complex models and longer training sequences. The two new algorithms have the added advantage of being easier to implement than the corresponding default algorithms for Viterbi training and stochastic EM training.

## Background

Hidden Markov models (HMMs) and their variants are widely used for analyzing biological sequence data. Bioinformatics applications range from methods for comparative gene prediction (e.g. [1,2]) to methods for modeling promoter grammars (e.g. [3]), identifying protein domains (e.g. [4]), predicting protein interfaces (e.g. [5]), the topology of transmembrane proteins (e.g. [6]) and residue-residue contacts in protein structures (e.g. [7]), querying pathways in protein interaction networks (e.g. [8]), predicting the occupancy of transcription factors (e.g. [9]) as well as inference models for genome-wide association studies (e.g. [10]) and disease association tests for inferring ancestral haplotypes (e.g. [11]).

Most of these bioinformatics applications have been set up for a specific type of analysis and a specific biological data set, at least initially. The states of the underlying HMM and the implemented prediction algorithms determine which type of data analysis can be performed, whereas the parameter values of the HMM are chosen for a particular data set in order to optimize the corresponding prediction accuracy. If we want to apply the same method to a new data set, e.g. predict genes in a different genome, we need to adjust the parameter values in order to make sure the performance accuracy is optimal.

Manually adjusting the parameters of an HMM in order to get a high prediction accuracy can be a very time consuming task which is also not guaranteed to improve the performance accuracy. A variety of training algorithms have therefore been devised in order to address this challenge. These training algorithms require as input and starting point a so-called *training set *of (typically partly annotated) data. Starting with a set of (typically user-chosen) initial parameter values, the training algorithm employs an iterative procedure which subsequently derives new, more refined parameter values. The iterations are stopped when a termination criterion is met, e.g. when a maximum number of iterations have been completed or when the change of the log-likelihood from one iteration to the next become sufficiently small. The model with the final set of parameters is then used to test if the performance accuracy has been improved. This is typically done by analyzing a *test set *of annotated data which has no overlap with the training set by comparing the predicted to the known annotation.

Of the training algorithms used in bioinformatics applications, the Viterbi training algorithm [12,13] is probably the most commonly used, see e.g. [14-16]. This is due to the fact that it is easy to implement if the Viterbi algorithm [17] is used for generating predictions. In each iteration of Viterbi training, a new set of parameter values *ϕ *is derived from the counts of emissions and transitions in the Viterbi paths Π^∗ ^for the set of training sequences X. Because the new parameters are completely determined by the Viterbi paths, Viterbi training converges as soon as the Viterbi paths no longer change or, alternatively, if a fixed number of iterations have been completed. Viterbi training finds at best a local optimum of the likelihood *P*(X, Π^∗^|*ϕ*), i.e. it derives parameter values *ϕ *that maximize the contribution from the set of Viterbi paths Π^∗ ^to the likelihood. There already exist a number of algorithms that can make Viterbi decoding computationally more efficient. Keibler *et al*. [18] introduce two heuristic algorithms for Viterbi decoding which they implement into the gene-prediction program TWINSCAN/N-SCAN, called "Treeterbi" and "Parallel Treeterbi", which have the same worst case asymptotic memory and time requirements as the standard Viterbi algorithm, but which in practice work in a significantly more memory efficient way. Sramek *et al*. [19] present a new algorithm, called "on-line Viterbi algorithm" which renders Viterbi decoding more memory efficient without significantly increasing the time requirement. The most recent contribution is from Lifshits *et al*. [20] who propose more efficient algorithms for Viterbi decoding and Viterbi training. These new algorithms exploit repetitions in the input sequences (in five different ways) in order to accelerate the default algorithm.

Another well-known training algorithm for HMMs is Baum-Welch training [21] which is an expectation maximization (EM) algorithm [22]. In each iteration, a new set of parameter values is derived from the estimated number of counts of emissions and transitions by considering *all *possible state paths (rather than only a single Viterbi path) for every training sequence. The iterations are typically stopped after a fixed number of iterations or as soon as the change in the log-likelihood is sufficiently small. For Baum-Welch training, the likelihood *P*(X|*ϕ*) [13] can be shown to converge (under some conditions) to a stationary point which is either a local optimum or a saddle point. Baum-Welch training using the traditional combination of forward and backward algorithm [13] is, for example, implemented into the prokaryotic gene prediction method EASYGENE [23] and the HMM-compiler HMMoC [15]. As for Viterbi training, the outcome of Baum-Welch training may strongly depend on the chosen set of initial parameter values. As Jensen [24] and Khreich *et al*. [25] describe, computationally more efficient algorithms for Baum-Welch training which render the memory requirement independent of the sequence length have been proposed, first in the communication field by [26-28] and later, independently, in bioinformatics by Miklós and Meyer [29], see also [30]. The advantage of this linear-memory memory algorithm is that it is comparatively easy to implement as it requires only a one- rather than a two-step procedure and as it scans the sequence in a uni- rather than bi-directional way. This algorithm was employed by Hobolth and Jensen [31] for comparative gene prediction and has also been implemented, albeit in a modified version, by Churbanov and Winters-Hilt [30] who also compare it to other implementations of Viterbi and Baum-Welch training including checkpointing implementations.

Stochastic expectation maximization (EM) training or Monte Carlo EM training [32] is another iterative procedure for training the parameters of HMMs. Instead of considering only a *single *Viterbi state path for a given training sequence as in Viterbi training or *all *state paths as in Baum-Welch training, stochastic EM training considers a fixed-number of *K *state paths Π*^s ^*which are sampled from the posterior distribution *P*(Π|*X*) for every training sequence *X *in every iteration. Sampled state paths have already been used in several bioinformatics applications for sequence decoding, see e.g. [2,33] where sampled state paths are used in the context of gene prediction to detect alternative splice variants.

All three above training algorithms, i.e. Viterbi training, Baum-Welch training and stochastic EM training, can be combined with the traditional check-pointing algorithm [34-36] in order to trade time for memory requirements.

We here introduce two new algorithms that make Viterbi training and stochastic EM training computationally more efficient. Both algorithms have the significant advantage of rendering the memory requirement independent of the sequence length for HMMs while keeping the time requirement the same (for Viterbi training) or modifying it by a factor of *M K*/(*M *+ *K*), i.e. decreasing it when only one state path *K *= 1 is sampled for a model of *M *states (for stochastic EM training). Both algorithms are inspired by the linear-memory algorithm for Baum-Welch training which requires only a uni-directional rather than bi-directional movement along the input sequence and which has the added advantage of being considerably easier to implement. We present a detailed description of the two new algorithms for Viterbi training and stochastic EM training. In addition, we implement all three algorithms, i.e. the new algorithms for Viterbi training and stochastic EM training and the previously published linear-memory algorithm for Baum-Welch training, into our HMM-compiler HMM-CONVERTER[37] and examine the practical features of these these three algorithms for three small example HMMs.

## Methods and Results

### Definitions and notation

In order to simplify the notation in the following, we will assume without loss of generality that we are dealing with a 1st-order HMM where the *Start *state and the *End *state are the only silent states. Our description of the existing and the new algorithms easily generalize to higher-order HMMs, HMMs with more silent states (provided there exists no circular path in the HMM involving only silent states) and *n*-HMMs, i.e. HMMs which read *n *un-aligned input sequences rather than a single input sequence at a time. An HMM is defined by

• a set of states S = {0, 1, ... , *M*}, where state 0 denotes the *start *and state *M *denotes the *End *state and where all other states are non-silent,

• a set of transition probabilities T = {*t*_*i*,*j *_|*i*, *j *∈ S}, where *t*_*i*,*j *_denotes the transition probability to go from state *i *to state *j *and $\sum_{j\in S}t_{i,j}=1$ for every state *i *∈ S and

• a set of emission probabilities ℰ = {*e*_*i*_(*y*)|*i *∈ S, *y *∈ A}, where *e*_*i*_(*y*) denotes the emission probability of state *i *for symbol *y *and $\sum_{y\in A}e_{i}(y)=1$ for every non-silent state *i *∈ S and A denotes the alphabet from which the symbols in the input sequences are derived, e.g. A = {A, C, G, T} when dealing with DNA sequences.

We also define:

• *T*_*max *_is the maximum number of states that any state in the model is connected to, also called the model's connectivity.

• X = {*X*^1^, *X*^2^, ... , *X^N ^*} denotes the training set of *N *sequences, where each particular training sequence *X^i ^*of length *L^i ^*is denoted $X^{i}=(x_{1}^{i},x_{2}^{i},\ldots ,x_{L^{i}}^{i})$. In the following and to simplify the notation, we pick one particular training sequence *X *∈ X of length *L *as representative which we denote *X *= (*x*_1_, *x*_2_, ..., *x*_*L*_). We write *X*_*n *_= (*x*_1_, *x*_2_, ... , *x*_*n*_), *n *∈ {1, ... , *L*}, to denote the sub-sequence of *X *which finishes at sequence position *n*.

• Π = (*π*_0_, *π*_1_, ... , *π*_*L*+1_) denotes a state path in the HMM for an input sequence *X *of length *L*, i.e. state *π*_*i *_is assigned to sequence position *x*_*i*_. Π^∗ ^denotes a Viterbi path and Π*^s ^*a state path that has been sampled from the posterior distribution *P*(Π|*X *) of the corresponding sequence *X*.

### A linear-memory algorithm for Viterbi training

Of the HMM-based methods that provide automatic algorithms for parameter training, Viterbi training [13] is the most popular. This is primarily due to the fact that Viterbi training is readily implemented if the Viterbi algorithm is used to generate predictions. Similar to Baum-Welch training [21,22], Viterbi training is an iterative training procedure. Unlike Baum-Welch training, however, which considers *all *state paths for a given training sequence in each iteration, Viterbi training only considers a *single *state path, namely a Viterbi path, when deriving new sets of parameters. In each iteration, a new set of parameter values is derived from the counts of emissions and transitions in the Viterbi paths [17] of the training sequences. The iterations are terminated as soon as the Viterbi paths of the training sequences no longer change.

In the following,

• let $E_{i}^{q}(y,X,\Pi^{*}(X))$ denote the number of times that state *i *reads symbol *y *from input sequence *X *in Viterbi path Π^∗^(*X*) given the HMM with parameters from the *q*-th iteration,

• in particular let $E_{i}^{q}(y,X_{k},\Pi^{*}(X_{k},\pi_{k}^{*}=m))$ denote the number of times that state *i *reads symbol *y *from input sequence *X *in the partial Viterbi path $\Pi^{*}(X_{k},\pi_{k}^{*}=m)=(\pi_{0}^{*},\ldots ,\pi_{k-1}^{*},\pi_{k}^{*}=m)$ which finishes at sequence position *k *in state *m*, and

• let $T_{i,j}^{q}(X,\Pi^{*}(X))$ denote the number of times that a transition from state *i *to state *j *is used in Viterbi path Π^∗^(*X*) for sequence *X *given the HMM with parameters from the *q*-th iteration,

• in particular let $T_{i,j}^{q}(X_{k},\Pi^{*}(X_{k},\pi_{k}^{*}=m))$ denote the number of times that a transition from state *i *to state *j *is used in the partial Viterbi path $\Pi^{*}(X_{k},\pi_{k}^{*}=m)=(\pi_{0}^{*},\ldots ,\pi_{k-1}^{*},\pi_{k}^{*}=m)$ which finishes at sequence position *k *in state *m*.

In the following, the superscript *q *will indicate from which iteration the underlying parameters derive. If we consider all *N *sequences of a training set X = {*X*^1^, ... *X^N ^*} and a Viterbi path Π^∗^(*X^n^*) for each sequence *X^n ^*in the training set, the recursion which updates the values of the transition and emission probabilities reads:

(1) $$t_{i,j}^{q+1}=\frac{\sum_{n=1}^{N}T_{i,j}^{q}(X^{n},\Pi^{*}(X^{n}))}{\sum_{j=1}^{M}\sum_{n=1}^{N}T_{i,j}^{q}(X^{n},\Pi^{*}(X^{n}))}$$

(2) $$e_{i}^{q+1}(y)=\frac{\sum_{n=1}^{N}E_{i}^{q}(y,X^{n},\Pi^{*}(X^{n}))}{\sum_{y'\in A}\sum_{n=1}^{N}E_{i}^{q}(y',X^{n},\Pi^{*}(X^{n}))}$$

These equations assume that we know the values of $T_{i,j}^{q}(X^{n},\Pi^{*}(X^{n}))$ and $E_{i}^{q}(y,X^{n},\Pi^{*}(X^{n}))$, i.e. how often each transition and emission is used in the Viterbi path Π^∗^(*X^n^*) for training sequence *X^n^*.

One straightforward way to determine $T_{i,j}^{q}(X^{n},\Pi^{*}(X^{n}))$ and $E_{i}^{q}(y,X^{n},\Pi^{*}(X^{n}))$ is to first calculate the two-dimensional Viterbi matrix for every training sequence *X^n^*, to then derive a Viterbi state path Π^∗^(*X^n^*) from each Viterbi matrix using the well-known traceback procedure [17] and to then simply count how often each transition and each emission was used. Using this strategy, every iteration in the Viterbi training algorithm would require O(*M *max_*i*_{*L*_*i*_} + max_*i*_{*L*_*i*_}) memory and $O(MT_{max}\sum_{i=1}^{N}L_{i}+\sum_{i=1}^{N}L_{i})$ time, where $\sum_{i=1}^{N}L_{i}$ is the sum of the *N *sequence lengths in the training set X and max_*i*_{*L*_*i*_} the length of the longest sequence in training set X. However, for many bioinformatics applications where the number of states in the model *M *is large, the connectivity *T*_*max *_of the model high or the training sequences are long, these memory and time requirements are too large to allow automatic parameter training using this algorithm.

A linear-memory version of the Viterbi algorithm, called the Hirschberg algorithm [38], has been known since 1975. It can be used to derive Viterbi paths in memory that is linearized with respect to the length of one of the input sequences while increasing the time requirement by at most a factor of two. The Hirschberg algorithm, however, only applies to *n*-HMMs with *n *≥ 2, i.e. HMMs which read two or more un-aligned input sequences at a time. One significant disadvantage of the Hirschberg algorithm is that it is considerably more difficult to implement than the Viterbi algorithm. Only few HMM-based applications in bioinformatics actually employ it, see e.g. [1,37,39]. We will see in the following how we can devise a linear-memory algorithm for Viterbi training that does not involve the Hirschberg algorithm and that can be applied to all *n*-HMMs including *n *= 1.

We now introduce a linear-memory algorithm for Viterbi training. The idea for this algorithm stems from the following observations:

(V1) If we consider the description of the Viterbi algorithm [17], in particular the recursion, we realize that the calculation of the Viterbi values can be continued by retaining only the values for the previous sequence position.

(V2) If we have a close look at the description of the traceback procedure [17], we realize that we only have to remember the Viterbi matrix elements at the *previous *sequence position in order to deduce the state from which the Viterbi matrix element at the *current *sequence position and state was derived.

(V3) If we want to derive the Viterbi path Π from the Viterbi matrix, we have to start at the end of the sequence in the *End *state *M*.

Observations (V1) and (V2) imply that local information suffices to continue the calculation of the Viterbi matrix elements (V1) and to derive a previous state (V2) if we already are in a particular state and sequence position, whereas observation (V3) reminds us that in order to derive the Viterbi path, we have to start at the *end *of the training sequence. Given these three observations, it is not obvious how we can come up with a computationally more efficient algorithm for training with Viterbi paths. In order to realize that a more efficient algorithm exists, one also has to also note that:

(V4) While calculating the Viterbi matrix elements in the memory-efficient way outlined in (V1), we can *simultaneously *keep track of the previous state from which the Viterbi matrix element at every current state and sequence position was derived. This is possible because of observation (V2) above.

(V5) In every iteration *q *of the training procedure, we only need to know the values of $T_{i,j}^{q}(X,\Pi^{*}(X))$ and $E_{i}^{q}(y,X,\Pi^{*}(X))$, i.e. how *often *each transition and emission was used in each Viterbi state path Π^∗^(*X*) for every training sequence *X *, but not *where *in the Viterbi matrix each transition and emission was used.

Given all observations (V1) to (V5), we can now formally write down an algorithm which calculates $T_{i,j}^{q}(X,\Pi^{*}(X))$ and $E_{i}^{q}(y,X,\Pi^{*}(X))$ in a computationally efficient way which linearizes the memory requirement with respect to the sequence length and which is also easy to implement. In order to simplify the notation, we describe the following algorithm for one particular training sequence *X *and omit the superscript for the iteration *q*, as both remain the same throughout the algorithm. In the following,

• *T*_*i*,*j *_(*k*, *m*) denotes the number of times the transition from state *i *to state *j *is used in a Viterbi state path that finishes at sequence position *k *in state *m*,

• *E*_*i*_(*y*, *k*, *m*) denotes the number of times that state *i *reads symbol *y *in a Viterbi state path that finishes at sequence position *k *in state *m*,

• *v*_*i*_(*k*) denotes the Viterbi matrix element for state *i *and sequence position *k*, i.e. *v*_*i*_(*k*) is the probability of the Viterbi state path, i.e. the state path with the highest overall probability, that starts at the beginning of the sequence in the *Start *state and finishes in state *i *as sequence position *k*,

• *i*, *j*, *n *∈ S, *y *∈ A and *l *∈ S denotes the previous state from which the current Viterbi matrix element *v*_*m*_(*k*) was derived, and

• *δ*_*i*,*j *_is the delta-function with *δ*_*i*,*j *_= 1 for *i *= *j *and *δ*_*i*,*j *_= 0 else.

**Initialization**: at the start of training sequence *X *= (*x*_1_,..., *x*_*L*_) and for all *m *∈ S, set

$$\begin{array}{c} v_{m}(0)=\{\begin{array}{cc} 1 & m=0\\ 0 & m\neq 0 \end{array}\\ T_{i,j}(0,\;m)=0\\ E_{i}(y,\;0,\;m)=0 \end{array}$$

**Recursion**: loop over all positions *k *from 1 to *L *in the training sequence *X *and loop, for each such sequence position *k*, over all states *m *∈ S\{0} = {1,..., *M *} and set

$$\begin{array}{c} v_{m}(k)=e_{m}(x_{k})\cdot \underset{n\in S}{\text{max}}\{v_{n}(k-1)\cdot t_{n,m}\}\\ T_{i,j}(k,m)=T_{i,j}(k-1,l)+\delta_{l,i}\cdot \delta_{m,j}\\ E_{i}(y,k,m)=E_{i}(y,k-1,l)+\delta_{m,i}\cdot \delta_{y,x_{k}} \end{array}$$

where *l *denotes the state at the previous sequence position *k − *1 from which the Viterbi matrix element *v*_*m*_(*k*) for state *m *and sequence position *k *derives, i.e. $l=\arg\max_{n\in S}\{v_{n}(k-1)\cdot t_{n,m}\}$.

**Termination**: at the end of the input sequence, i.e. for *k *= *L *and for *m *= *M *the silent *End *state, set

$$\begin{array}{c} v_{M}(L)=\underset{n\in S}{\text{max}}\{v_{n}(L)\cdot t_{n,M}\}\\ T_{i,j}(L,M)=T_{i,j}(L,l)+\delta_{l,i}\cdot \delta_{M,j}\\ E_{i}(y,L,M)=E_{i}(y,L,l) \end{array}$$

where *l *denotes the state at the sequence position *L *from which the Viterbi matrix element *v*_*M *_(*L*) for the *End *state *M *and sequence position *L *derives, i.e. $l=\arg\max_{n\in S}\{v_{n}(L)\cdot t_{n,M}\}$.

The above algorithm yields $T_{i,j}(L,M)=T_{i,j}^{q}(X,\Pi^{*}(X))$ and $E_{i}(y,L,M)=E_{i}^{q}(y,X,\Pi^{*}(X))$ (and *v*_*M*_(*L*) =*P^q^*(*X*, Π^∗^(*X*))), i.e. we know how often a transition from state *i *to state *j *was used and how often symbol *y *was read by state *i *in Viterbi state path Π^∗^(*X*) in iteration *q*.

**Theorem 1: **The above algorithm yields $T_{i,j}(L,M)=T_{i,j}^{q}(X,\Pi^{*}(X))$ and $E_{i}(y,L,M)=E_{i}^{q}(y,X,\Pi^{*}(X))$.

**Proof: **We will prove these statements via induction with respect to the sequence position *k*.

**(1) Induction start at ***k *= 0: This corresponds to the initialization step in the algorithm. *T*_*i*,*j *_(0, *m*) = 0 and *E*_*i*_(*y*, 0, *m*) = 0 for all *m *∈ S as any zero-length Viterbi path finishing in state *m *at sequence position 0 has zero transitions from state *i *to *j *and has not read any sequence symbol.

**(2) Induction step ***k *− 1 → *k ***for ***k *∈ {1,...*L *− 1} **if the state at sequence position ***k *= *L ***is not the *End *state ***M *: This case corresponds to the recursion in the algorithm. We assume that $T_{i,j}(k-1,m)=T_{i,j}^{q}(X_{k-1},\Pi^{*}(X_{k-1},\pi_{k-1}^{*}=m))$ and $E_{i}(y,k-1,m)=E_{i}^{q}(y,X_{k-1},\Pi^{*}(X_{k-1},\pi_{k-1}^{*}=m))$.

We need to distinguish two cases (a) and (b). Let *l *denote the state at sequence position *k *− 1 from which the Viterbi matrix element *v*_*m*_(*k*) for state *m *and sequence position *k *derives, i.e. $l=\arg\max_{n\in S}\{v_{n}(k-1)\cdot t_{n,m}\}$.

• **Case (a):**

**Emissions (i): ***m *= *i ***and ***y *= *x*_*k *_: In this case, *E*_*i*_(*y*, *k*, *m*) = *E*_*i*_(*y*, *k *− 1, *l*) + 1. As we know that *E*_*i*_(*y*, *k *− 1, *l*) is the number of times that state *i *reads symbol *y *in a Viterbi path ending in state *l *at sequence position *k − *1, we need to add 1 count for reading symbol *y *= *x*_*k *_by state *m *= *i *at the next sequence position *k *in order to obtain *E*_*i*_(*y*, *k*, *m*).

**Transitions (ii): ***l *= *i ***and ***m *= *j*: In this case, *T*_*i,j *_(*k*, *m*) = *T*_*i*,*j*_(*k − *1, *l*) + 1. As we know that *T*_*i*,*j *_(*k − *1, *l*) is the number of times that a transition from state *i *to state *j *is used in a Viterbi path ending in state *l *at sequence position *k − *1, we need to add 1 count for the transition from state *l *= *i *to state *m *= *j *which brings us from sequence position *k − *1 to *k *in order to get *T*_*i*,*j *_(*k*, *m*).

• **Case (b):**

**Emissions (i): ***m *≠ *i ***or ***y *≠ *x*_*k *_: In this case, *E*_*i*_(*y*, *k*, *m*) = *E*_*i*_(*y*, *k − *1, *l*). We know that *E*_*i*_(*y*, *k *− 1, *l*) is the number of times that state *i *reads symbol *y *in a Viterbi path ending in state *l *at sequence position *k − *1. If we go from state *l *at position *k − *1 to state *m *at position *k *and read symbol *x*_*k *_and if *m *≠ *i *or *y *≠ *x*_*k *_, we do not need to modify the number of counts as we know that state *i *at position *k *does not read symbol *y*, i.e. *E*_*i*_(*y*, *k*, *m*) = *E*_*i*_(*y*, *k − *1, *l*).

**Transitions (ii): ***l *≠ *i ***or ***m *≠ *j*: In this case, *T*_*i*,*j *_(*k*, *m*) = *T*_*i*,*j *_(*k − *1, *l*). We know that *T*_*i*,*j *_(*k *− 1, *l*) is the number of times that a transition from state *i *to state *j *is used in a Viterbi path ending in state *l *at sequence position *k *− 1. If we make a transition from state *l *at position *k − *1 to state *m *at position *k *and if *l *≠ *i *or *m *≠ *j*, we do not need to modify the number of counts as we know this is not a transition from state *i *to state *j*, i.e. *T*_*i*,*j *_(*k*, *m*) = *T*_*i*,*j *_(*k − *1, *l*).

**(3) If the state at sequence position ***k = L ***is the *End *state ***M *: This case corresponds to the termination step in the algorithm. As in (2), we need to distinguish two cases (a) and (b), but now only for the transition counts. Let *l *denote the state at sequence position *L *from which the Viterbi matrix element *v*_*M *_(*L*) for the *End *state *M *and sequence position *L *derives, i.e. $l=\arg\max_{n\in S}\{v_{n}(L)\cdot t_{n,m}\}$.

**Emissions (i): **In this case, *E*_*i*_(*y*, *L*, *M*) = *E*_*i*_(*y*, *L*, *l*). As we know that *E*_*i*_(*y*, *L*, *l*) is the number of times that state *i *reads symbol *y *in a Viterbi path ending in state *l *at sequence position *L*, we do not need to modify this number of counts when going to the silent *End *state at the same sequence position *L *as silent states do not read any symbols from the input sequence. As we are now at the end of the input sequence *X *and the Viterbi path Π^∗^(*X*), we have $E_{i}(y,L,M)=E_{i}^{q}(y,X,\Pi^{*}(X))$.

• **Case (a):**

**Transitions (i): ***l *= *i ***and ***M *= *j*: In this case, *T*_*i*,*j *_(*L*, *M*) = *T*_*i*,*j *_(*L*, *l*) + 1. As we know that *T*_*i*,*j *_(*L*, *l*) is the number of times that a transition from state *i *to state *j *is used in a Viterbi path ending in state *l *at sequence position *L*, we need to add 1 count for the transition from state *l *= *i *to the *End *state *M *= *j *at sequence position *L*. Note that this transition of state does not incur a change of sequence position as the *End *state is a silent state. As we are now at the end of the input sequence *X *and the Viterbi path Π^∗^(*X*), we have $T_{i,j}(L,M)=T_{i,j}^{q}(X,\Pi^{*}(X))$.

• **Case (b):**

**Transitions (i): ***l *≠ *i ***or ***M *≠ *j*: In this case, *T*_*i*,*j *_(*L*, *M *) = *T*_*i*,*j *_(*L*, *l*). We know that *T*_*i*,*j *_(*L*, *l*) is the number of times that a transition from state *i *to state *j *is used in a Viterbi path ending in state *l *at sequence position *L*. If we make a transition from state *l *at position *L *to the *End *state *M *at sequence position *L *and if *l *≠ *i *or *M *≠ *j*, we do not make a transition from state *i *to state *j *and thus do not need to modify the number of counts, i.e. *T*_*i*,*j *_(*L*, *M*) = *T*_*i*,*j *_(*L*, *l*). Also in case (a), we are now at the end of the input sequence *X *and the Viterbi path Π^∗^(*X *) and thus have $T_{i,j}(L,M)=T_{i,j}^{q}(X,\Pi^{*}(X)).$

**End of proof**.

As is clear from the above description of the algorithm, the calculation of the *v*_*m*_, *T*_*i*,*j *_and *E*_*i *_values for sequence position *k *requires only the respective values for the previous sequence position *k − *1, i.e. the memory requirement can be linearized with respect to the sequence length.

For an HMM with *M *states and a training sequence of length *L *and for every free parameter of the HMM that we want to train, we thus need in every iteration O(*M *) memory to store the *v*_*m *_values and O(*M*) memory to store the cumulative counts for the free parameter itself, e.g. the *T*_*i*,*j *_values for a particular transition from state *i *to state *j*. For an HMM, the memory requirement of the training using the new algorithm is thus independent of the length of the training sequence.

For training one free parameter in the HMM with the above algorithm, each iteration requires O(*MT*_*max*_*L*) time to calculate the *v*_*m *_values and to calculate the cumulative counts. If *Q *is the total number of free parameters in the model and if we choose *P *of these parameters to be trained in parallel, i.e. *P *∈ {1,...*Q*} and *Q*/*P *∈ ℕ, the memory requirement increases slightly to O(*MP *) and the time requirement becomes $O(MT_{max}L\frac{Q}{P})$. This algorithm can therefore be readily adjusted to trade memory and time requirements, e.g. to maximize speed by using the maximum amount of available memory. This can be directly compared to the default algorithm for Viterbi training described above with first calculates the entire Viterbi matrix and which requires O(*M L*) memory and O(*T*_*max*_*LM*) time to achieve the same. Our new algorithm thus has the significant advantage of linearizing the memory requirement with respect to the sequence length while keeping the time requirement the same, see Table 1 for a detailed overview. Our new algorithm is thus as memory efficient as Viterbi training using the Hirschberg algorithm, while being more time efficient, significantly easier to implement and applicable to all *n*-HMMs, including the case *n *= 1.

**Table 1:** Theoretical computational requirements

training one parameter at a time				
**type of training**	**algorithm**	**time**	**memory**	**reference**

Viterbi	Viterbi	O(*T*_*max*_*LM*)	O(*ML*)	[17]
	Lam-Meyer	O(*T*_*max*_*LM*)	O(*M*)	this paper

Baum-Welch	Baum-Welch	O(*T*_*max*_*LM*)	O(*ML*)	[13]
	checkpointing	O(*T*_*max*_*LM *log(*L*))	O(*M *log(*L*))	[34]
	linear-memory	O*(T*_*max*_*LM*)	O(*M*)	[29]

stochastic EM	forward & back-tracing	O(*T*_*max*_*L*(*M + K*))	O(*ML*)	[32]
	Lam-Meyer	O(*T*_*max*_*LMK*)	O(*MK + T*_*max*_)	this paper
training *P *of *Q *parameters at the same time with *P ***∈ **{1, ..., *Q*} and *Q/P ***∈ **ℕ				

Viterbi	Viterbi	O(*T*_*max*_*LMQ/P*)	O(*ML*)	[17]
	Lam-Meyer	O(*T*_*max*_*LMQ/P*)	O(*MP*)	this paper

Baum-Welch	Baum-Welch	O(*T*_*max*_*LMQ/P*)	O(*ML + P*)	[13]
	checkpointing	O(*T*_*max*_*LMQ *log(*L/P*))	O(*M *log(*L*))	[34]
	linear-memory	O(*T*_*max*_*LM Q/P*)	O (*M*)	[29]

stochastic EM	forward & back-tracing	O(*T*_*max*_*L*(*M *+ *K*)*Q/P *)	O(*ML*)	[32]
	Lam-Meyer	O(*T*_*max*_*LMKQ/P *)	O(*MKP + T*_*max*_)	this paper

Overview of the theoretical time and memory requirements for Viterbi training, Baum-Welch training and stochastic EM training for an HMM with *M *states, a connectivity of *T*_*max *_and *Q *free parameters. *K *denotes the number of state paths sampled in each iteration for every training sequence for stochastic EM training. The time and memory requirements below are the requirements per iteration for a single training sequence of length *L*. It is up to the user to decide whether to train the *Q *free parameters of the model sequentially, i.e. one at a time, or in parallel in groups. The two tables below cover all possibilities.

In the general case we are dealing with a training set X = {*X*^1^, *X*^2^, ... , *X^N^*} of *N *sequences, where the length of training sequence *X^i ^*is *L^i^*. If training involves the entire training set, i.e. all training sequences simultaneously, *L *in the formulae below needs to be replaced by $\sum_{i=1}^{N}L_{i}$ for the memory requirements and by max_*i*_{*L*_*i*_} for the time requirements. If, on the other hand, training is done by considering by one training sequence at a time, *L *in the formulae below needs to be replaced by $\sum_{i=1}^{N}L_{i}$ for the time requirements and by max_*i*_{*L*_*i*_} for the memory requirements.

### A linear-memory algorithm for stochastic EM training

One alternative to Viterbi training is Baum-Welch training [21], which is an expectation maximization (EM) algorithm [22]. As Viterbi training, Baum-Welch training is an iterative procedure. In each iteration of Baum-Welch training, the estimated number of counts for each transition and emission is derived by considering *all *possible state paths for a given training sequence in the model rather than only the single Viterbi path. As discussed in the introduction, there already exists an efficient algorithm for Baum-Welch training which linearizes the memory requirement with respect to the sequence length and which is also relatively easy to implement.

One variant of Baum-Welch training is called stochastic EM algorithm [32]. Unlike Viterbi training which considers only a *single *state path and unlike Baum-Welch training which considers *all *possible state paths for every training sequence, the stochastic EM algorithm derives new parameter values from a *fixed *number of *K *state paths (each of which is denoted Π*^s^*(*X*)) that are sampled for each training sequence from the posterior distribution *P*(Π|*X*). Similar to Viterbi and Baum-Welch training, the stochastic EM algorithm employs an iterative procedure. As for Baum-Welch training, the iterations are stopped once a maximum number of iterations have been reached or once the change in the log-likelihood is sufficiently small.

In strict analogy to the notation we introduced for Viterbi training, $E_{i}^{q}(y,X,\Pi^{s}(X))$ denotes the number of times that state *i *reads symbol *y *from input sequence *X *in a sampled state path Π*^s^*(*X*) given the HMM with parameters from the *q*-th iteration. Similarly, $T_{i,j}^{q}(X,\Pi^{s}(X))$ denotes the number of times that a transition from state *i *to state *j *is used in a sampled state path Π*^s^*(*X*) for sequence *X *given the HMM with parameters from the *q*-th iteration.

As usual, the superscript *q *indicates from which iteration the underlying parameters of the HMM derive. If we consider all *N *sequences of the training set X = {*X*^1^, ... *X^N^*} and sample *K *state paths $\Pi_{k}^{s}(X^{n})$, *k *∈ {1, ... *K*}, for each sequence *X^n ^*in the training set, the step which updates the values of the transition and emission probabilities can be written as:

$$\begin{array}{c} t_{i,j}^{q+1}=\frac{\sum_{n=1}^{N}\sum_{k=1}^{K}T_{i,j}^{q}(X^{n},\Pi_{k}^{s}(X^{n}))}{\sum_{j'=1}^{M}\sum_{n=1}^{N}\sum_{k=1}^{K}T_{i,j'}^{q}(X^{n},\Pi_{k}^{s}(X^{n}))}\\ e_{i}^{q+1}(y)=\frac{\sum_{n=1}^{N}\sum_{k=1}^{K}E_{i}^{q}(y,X^{n},\Pi_{k}^{s}(X^{n}))}{\sum_{y'\in A}\sum_{n=1}^{N}\sum_{k=1}^{K}E_{i}^{q}(y',X^{n},\Pi_{k}^{s}(X^{n}))} \end{array}$$

These expressions are strictly analogous to equations 1 and 2 that we introduced for Viterbi training. As before, these assume that we know the values of $T_{i,j}^{q}(X^{n},\Pi_{k}^{s}(X^{n}))$ and $E_{i}^{q}(y,X^{n},\Pi_{k}^{s}(X^{n}))$, i.e. how often each transition and emission is used in each sampled state path $\Pi_{k}^{s}(X^{n})$ for every training sequence *X^n^*.

#### Obtaining the counts from the forward algorithm and stochastic back-tracing

It is well-known that we can obtain the above counts *T*_*i*,*j *_(*X*, Π*^s^*(*X*)) and *E*_*i*_(*y*, *X*, Π*^s^*(*X*)) for a given training sequence *X*, iteration *q *and a sampled state path Π*^s^*(*X*) by using a combination of the forward algorithm and stochastic back-tracing [13,32]. For this, we first calculate all values in the two-dimensional forward matrix using the forward algorithm and then invoke the stochastic back-tracing procedure to sample a state-path Π*^s^*(*X*) from the posterior distribution *P*(Π|*X*).

We will now explain these two algorithms in detail in order to facilitate the introduction of our new algorithm. In the following,

• *f*_*i*_(*k*) denotes the sum of probabilities of all state paths that have read training sequence *X *up to and including sequence position *k *and that end in state *i*, i.e. *f*_*i*_(*k*) = *P*(*x*_1_, ..., *x*_*k *_, *s*(*x*_*k *_) = *i*), where *s*(*x*_*k*_) denotes the state that reads sequence position *x*_*k *_from input sequence *X*. We call *f*_*i*_(*k*) the forward probability for sequence position *k *and state *i*.

• *p*_*i*_(*k*, *m*) denotes the probability of selecting state *m *as the previous state while being in state *i *at sequence position *k *(i.e. sequence position *k *has already been read by state *i*), i.e. *p*_*i*_(*k*, *m*) = *P*(*π*_*k*−1 _= *m*|*π*_*k *_= *i*). For a given sequence position *k *and state *i*, *p*_*i*_(*k*, *m*) defines a probability distribution over previous states as $\sum_{m}p_{i}(k,\;m)=1$.

The forward matrix is calculated using the forward algorithm [13]:

**Initialization: **at the start of the input sequence, consider all states *m *∈ S in the model and set

$$f_{m}(0)=\{\begin{array}{c} 1\;m=0\\ 0\;m\neq 0 \end{array}$$

**Recursion**: loop over all positions *k *from 1 to *L *in the input sequence and loop, for each such sequence position *k*, over all states *m *∈ S\{0} = {1, ... , *M*} and set

(3) $$f_{m}(k)=e_{m}(x_{k})\cdot \sum_{n=0}^{M}f_{n}(k-1)\cdot t_{n,m}$$

**Termination**: at the end of the input sequence, i.e. for *k = L *and *m = M *the *End *state, set

$$P(X)=f_{M}(L)=\sum_{n=0}^{M}f_{n}(L_{x})\cdot t_{n,M}$$

Once we have calculated all forward probabilities *f*_*i*_(*k*) in the two-dimensional forward matrix, i.e. for all states *i *in the model and all positions *k *in the given training sequence *X*, we can then use the stochastic back-tracing procedure [13] to sample a state path from the posterior distribution *P*(Π|*X*).

The stochastic back-tracing starts at the end of the input sequence, i.e. at sequence position *k = L*, in the *End *state, i.e. *i *= *M *, and selects state *m *as the previous state with probability:

(4) $$p_{i}(k,\;m)=\{\begin{array}{ll} \frac{f_{m}(k-1)\cdot e_{i}(x_{k})\cdot t_{m,i}}{f_{i}(k)} & \text{if}\;\text{state}\;i\;\text{is}\;\text{not}\;\text{silent}\\ \frac{f_{m}(k)\cdot t_{m,i}}{f_{i}(k)} & \text{if}\;\text{state}\;i\;\text{is}\;\text{silent} \end{array}$$

This procedure is continued until we reach the start of the sequence and the *Start *state. The resulting succession of chosen previous states corresponds to one state path Π*^s^*(*X*) that was sampled from the posterior distribution *P*(Π|*X *).

The denominator in equation (4) corresponds to the sum of probabilities of all state paths that finish in state *i *at sequence position *k*, whereas the nominator corresponds to the sum of probabilities of all state paths that finish in state *i *at sequence position *k and *that have state *m *as the previous state.

When being in state *i *at sequence position *k*, we can therefore use this ratio to sample which previous state *m *we should have come from.

As this stochastic back-tracing procedure requires the entire matrix of forward values for all states and all sequence positions, the above algorithm for sampling a state path requires O(*ML*) memory and O(*MT*_*max*_*L*) time in order to first calculate the matrix of forward values and then O(*L*) memory and O(*LT*_*max*_) time for sampling a single state path from the matrix. Note that additional state paths can be sampled without having to recalculate the matrix of forward values. For sampling *K *state paths for the same sequence in a given iteration, we thus need O((*M *+ *K*)*T*_*max*_*L*) time and O(*ML*) memory, if we do not to store the sampled state paths themselves.

If our computer has enough memory to use the forward algorithm and the stochastic back-tracing procedure described above, each iteration in the training algorithm would require O(*M *max_*i*_{*L*_*i*_} + *K *max_*i*_{*L*_*i*_}) memory and $O(MT_{max}\sum_{i=1}^{N}L_{i}+K\sum_{i=1}^{N}L_{i})$ time, where $\sum_{i=1}^{N}L_{i}$ is the sum of the *N *sequence lengths in the training set X and max_*i*_{*L*_*i*_} the length of the longest sequence in training set X. As we do not have to keep the *K *sampled state paths in memory, the memory requirement can be reduced to O(*M *max_*i*_{*L*_*i*_}).

For many bioinformatics applications, however, where the number of states in the model *M *is large, the connectivity *T*_*max *_of the model high or the training sequences are long, these memory and time requirements are too large to allow automatic parameter training using stochastic EM training.

#### Obtaining the counts in a more efficient way

Our previous observations (V1) to (V5) that led to the linear-memory algorithm for Viterbi training can be replaced by similar observations for stochastic EM training:

(S1) If we consider the description of the forward algorithm above, in particular the recursion in Equation (3), we realize that the calculation of the forward values can be continued by retaining only the values for the previous sequence position.

(S2) If we have a close look at the description of the stochastic back-tracing algorithm, in particular the sampling step in Equation (4), we observe that the sampling of a previous state only requires the forward values for the current and the previous sequence position. So, provided we are at a particular sequence position and in a particular state, we can sample the state at the previous sequence position, if we know all forward values for the previous sequence position.

(S3) If we want to sample a state path Π*^s^*(*X*) from the posterior distribution *P*(Π|*X*), we have to start at the *end *of the sequence in the *End *state, see the description above and Equation (4) above. (The only valid alternative for sampling state paths from the posterior distribution would be to use the backward algorithm [13] instead of the forward algorithm and to then start the stochastic back-tracing procedure at the *start *of the sequence in the *Start *state.)

Observations (S1) and (S2) above imply that local information suffices to continue the calculation of the forward values (S1) and to sample a previous state (S2) if we already are in a particular state and sequence position, whereas observation (S3) reminds us that in order to sample from the correct probability distribution, we have to *start *the sampling at the *end *of the training sequence. Given these three observations, it is -- as before for Viterbi training -- not obvious how we can come up with a computationally more efficient algorithm. In order to realize that a more efficient algorithm does exist, one also has to note that:

(S4) While calculating the forward values in the memory-efficient way outlined in (S1) above, we can *simultaneously *sample a previous state for every combination of a state and a sequence position that we encounter in the calculating of the forward values. This is possible because of observation (S2) above.

(S5) In every iteration *q *of the training procedure, we only need to know the values of $T_{i,j}^{q}(X,\Pi^{s}(X))$ and $E_{i}^{q}(y,X,\Pi^{s}(X))$, i.e. how *often *each transition and emission appears in each sampled state path Π*^s^*(*X*) for every training sequence *X *, but not *where *in the matrix of forward values the transition or emission was used.

Given all observations (S1) to (S5) above, we can now formally write down a new algorithm which calculates $T_{i,j}^{q}(X,\Pi^{s}(X))$ and $E_{i}^{q}(y,X,\Pi^{s}(X))$ in a computationally more efficient way. In order to simplify the notation, we consider one particular training sequence *X *= (*x*_1_, ... *x*_*L*_) of length *L *and omit the superscript for the iteration *q*, as both remain the same throughout the following algorithm. In the following, *T*_*i*,*j *_(*k*, *m*) denotes the number of times the transition from state *i *to state *j *is used in a sampled state path that finishes at sequence position *k *in state *m *and *E*_*i*_(*y*, *k*, *m*) denotes the number of times state *i *read symbol *y *in a sampled state path that finishes at sequence position *k *in state *m*. As defined earlier, *f*_*i*_(*k*) denotes the forward probability for sequence position *k *and state *i*, *p*_*i*_(*k*, *m*) is the probability of selecting state *m *as the previous state while being in state *i *at sequence position *k*, *i*, *j*, *n *∈ S and *y *∈ A.

**Initialization**: at the start of the training sequence *X *and for all states *m *∈ S, set

$$\begin{array}{c} f_{m}(0)=\{\begin{array}{c} 1\;m=0\\ 0\;m\neq 0 \end{array}\\ T_{i,j}(0,m)=0\\ E_{i}(y,0,m)=0 \end{array}$$

**Recursion**: loop over all positions *k *from 1 to *L *in the training sequence *X *and loop, for each such sequence position *k*, over all states *m *∈ S\{0} = {1, ... , *M*} and set

$$\begin{array}{c} f_{m}(k)=e_{m}(x_{k})\cdot \sum_{n=0}^{M}f_{n}(k-1)\cdot t_{n,m}\\ p_{m}(k,n)=\frac{e_{m}(x_{k})\cdot f_{n}(k-1)\cdot t_{n,m}}{f_{m}(k)}\\ T_{i,j}(k,m)=T_{i,j}(k-1,\;l)+\delta_{l,i}\cdot \delta_{m,j}\\ E_{i}(y,k,m)=E_{i}(y,k-1,l)+\delta_{m,i}\cdot \delta_{y,x_{k}} \end{array}$$

where *l *denotes the state at previous sequence position *k − *1 that was sampled from the probability distribution *p*_*m*_(*k*, *n*), *n *∈ *S*, while being in state *m *at sequence position *k*.

**Termination**: at the end of the input sequence, i.e. for *k = L *and *m = M *the *End *state, set

$$\begin{array}{c} f_{M}(L)=\sum_{n=0}^{M}f_{n}(L_{x})\cdot t_{n,M}\\ p_{M}(L,n)=\frac{f_{n}(L)\cdot t_{n,M}}{f_{M}(L)}\\ T_{i,j}(L,M)=T_{i,j}(L,l)+\delta_{l,i}\cdot \delta_{M,j}\\ E_{i}(y,L,M)=E_{i}(y,L,l) \end{array}$$

where *l *now denotes the state at sequence position *L *that was sampled from the probability distribution *p*_*M*_(*L*, *n*), *n *∈ S, while being in the *End *state *M *at sequence position *L*, i.e. at the end of the training sequence.

The above algorithm yields $T_{i,j}(L,M)=T_{i,j}^{q}(X,\Pi^{s}(X))$, and $E_{i}(y,L,M)=E_{i}^{q}(y,X,\Pi^{s}(X))$ (and $f_{M}(L)=P^{q}(X))$, i.e. we know how often a transition from state *i *to state *j *was used and how often symbol *y *was read by state *i *in a state path Π*^S^*(*X*) sampled from the posterior distribution *P*(*X*|Π) in iteration *q *for sequence *X*.

**Theorem 2: **The above algorithm yields $T_{i,j}(L,M)=T_{i,j}^{q}(X,\Pi^{s}(X))$ and $E_{i}(y,L,M)=E_{i}^{q}(y,X,\Pi^{s}(X))$.

**Proof: **The proof for this theorem is very similar to the proof of theorem 1 for Viterbi training and therefore omitted. The key differences are, first, that *l *here corresponds to the state at the previous sequence position that is *sampled from a probability distribution *rather than deterministically determined and, second, that Π^*s *^here corresponds to a sampled state path rather than a deterministically derived Viterbi path Π*.

**End of proof**.

As is clear from the above algorithm, the calculation of the *f*_*m*_, *p*_*m*_, *T*_*i*,*j *_and *E*_*i *_values for sequence position *k *requires only the respective values for the previous sequence position *k − *1, i.e. the memory requirement can be linearized with respect to the sequence length.

For an HMM with *M *states, a training sequence of length *L *and for every free parameter to be trained, we thus need O(*M*) memory to store the *f*_*m *_values, O(*T*_*max*_) memory to store the *p*_*m *_values and O(*M*) memory to store the cumulative counts for the free parameter itself in every iteration, e.g. the *T*_*i*,*j *_values for a particular transition from state *i *to state *j*. If we sample *K *state paths, we have to store the cumulative counts from different state paths *separately*, i.e. we need *K *times more memory to store the cumulative counts for each free parameter, but the memory for storing the *f*_*m *_and the *p*_*m *_values remains the same. Overall, if *K *state paths are being sampled in each iteration, we thus need O(*M*) memory to store the *f*_*m *_values, O(*T*_*max*_) memory to store the *p*_*m *_values and O(*MK*) memory to store the cumulative counts for the free parameter itself in every iteration. For an HMM, the memory requirement of the new training algorithm is thus independent of the length of the training sequence.

For training one free parameter in the HMM with the above algorithm, each iterations requires O(*MT*_*max*_*L*) time to calculate the *f*_*m *_and the *p*_*m *_values and to calculate the cumulative counts for one training sequence. If *K *state paths are being sampled in each iteration, the time required to calculate the cumulative counts increases to O(*MT*_*max*_*LK*), but the time requirements for calculating the *f*_*m *_and *p*_*m *_values remains the same.

For sampling *K *state paths for the same input sequence and training one free parameter, we thus need O (*MK + T*_*max*_) memory and O(*MT*_*max*_*LK*) time for every iteration. If the model has *Q *parameters and if *P *of these parameters are to be trained in parallel, i.e. *P *∈ {1,...*Q*} and *Q/P *∈ ℕ, the memory requirement increases slightly to O(*MKP + T*_*max*_) and the time requirement becomes $O(MT_{max}LK\frac{Q}{P})$. As for Viterbi training, the linear-memory algorithm for stochastic EM training can therefore be readily used to trade memory and time requirements, e.g. to maximize speed by using the maximum amount of available memory, see Table 1 for a detailed overview.

This can be directly compared to the algorithm described in 2.1 with requires O(*ML*) memory and O(*T*_*max*_*L(M + K*)) time to do the same. Our new algorithm thus has the significant advantage of linearizing the memory requirement and making it independent of the sequence length for HMMs while increasing the time requirement only by a factor of $\frac{MK}{M+K}$, i.e. decreasing it when only one state path *K *= 1 is sampled.

### Examples

The algorithms that we introduce here can be used to train any HMM. The previous sections discuss the theoretical properties of the different parameter training methods in detail which are summarized in Table 1.

Even though the theoretical properties of the respective algorithms are independent of any particular HMM, the outcome of the different types of parameter training in terms of prediction accuracy and parameter convergence may very well depend on the features of a particular HMM. This is because the quantities that can be shown to be (locally) optimized by some training algorithms do not necessarily translate into an optimized prediction accuracy as defined by us here.

In order to investigate how well the different methods do in practice in terms of prediction accuracy and parameter convergence, we implemented Viterbi training, Baum-Welch training and stochastic EM training for three small example HMMs. For each model, we implemented the linear-memory algorithm for Baum-Welch training published earlier as well as the linear-memory algorithms for Viterbi training and stochastic EM training presented here.

In the first step, we use each model with the original parameter values to generate the sequences of the data set. We then randomly choose initial parameter values to initialize the HMM for parameter training. Each type of parameter training is performed three times using 2/3 of the un-annotated data set as training set and the remaining 1/3 of the data set for performance evaluation, i.e. we perform three cross-evaluation experiments for each model.

#### Example 1: The dishonest casino

**Figure 1:**
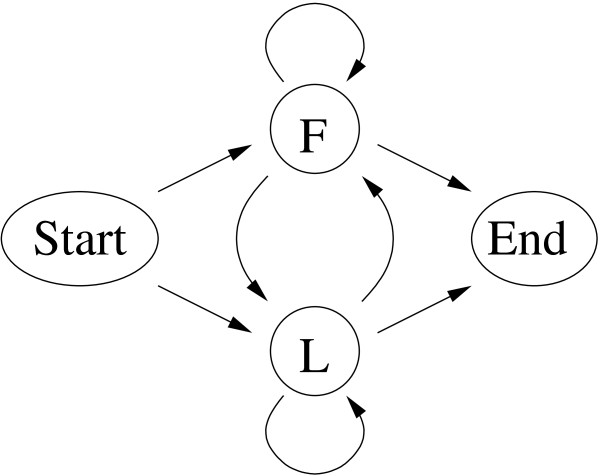
**HMM of the dishonest casino**. Symbolic representation of the HMM of the dishonest casino. States are shown as circles, transitions are shown as directed arrows. Please refer to the text for more details.

As first case, we consider the well-known example of the dishonest casino [13], see Figure 1. This casino consists of a fair (state F) and a loaded dice (state L). The fair dice generates numbers from A = {1, 2, 3, 4, 5, 6} with equal probability, whereas the loaded dice generates the same numbers in a biased way. The properties of the dishonest casino are readily captured in a four-state HMM with 8 transition and 12 emission probabilities, six each for each non-silent state *F *and *L*. Parameterizing the emission and transition probabilities of this HMM results in two independent transition probabilities and 10 independent emission probabilities, i.e. altogether 12 values to be trained. In order to avoid premature termination of parameter training, we use pseudo-counts of 1 for every parameter to be trained.

**Figure 2:**
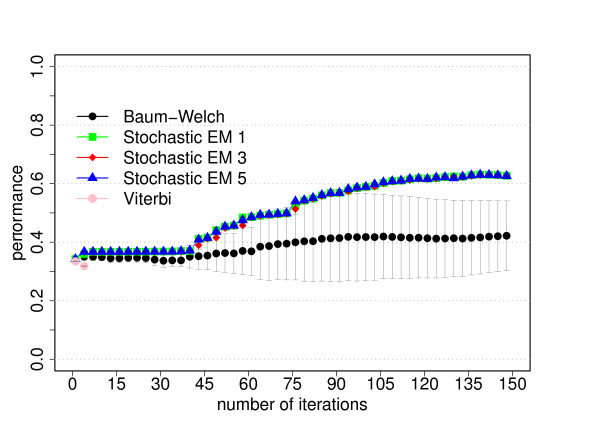
**Performance for the dishonest casino**. The average performance as function of the number of iterations for each training algorithm. The performance is defined as the product of the sensitivity and specificity and the average is the average of three cross-evaluation experiments. For stochastic EM training, a fixed number of state paths were sampled for each training sequence in each iteration (stochastic EM 1: one sampled state path, stochastic EM 3: three sampled state paths, stochastic EM 5: five sampled state paths). The error bars correspond to the standard deviation of the performance from the three cross-evaluation experiments. Please refer to the text for more information.

**Figure 3:**
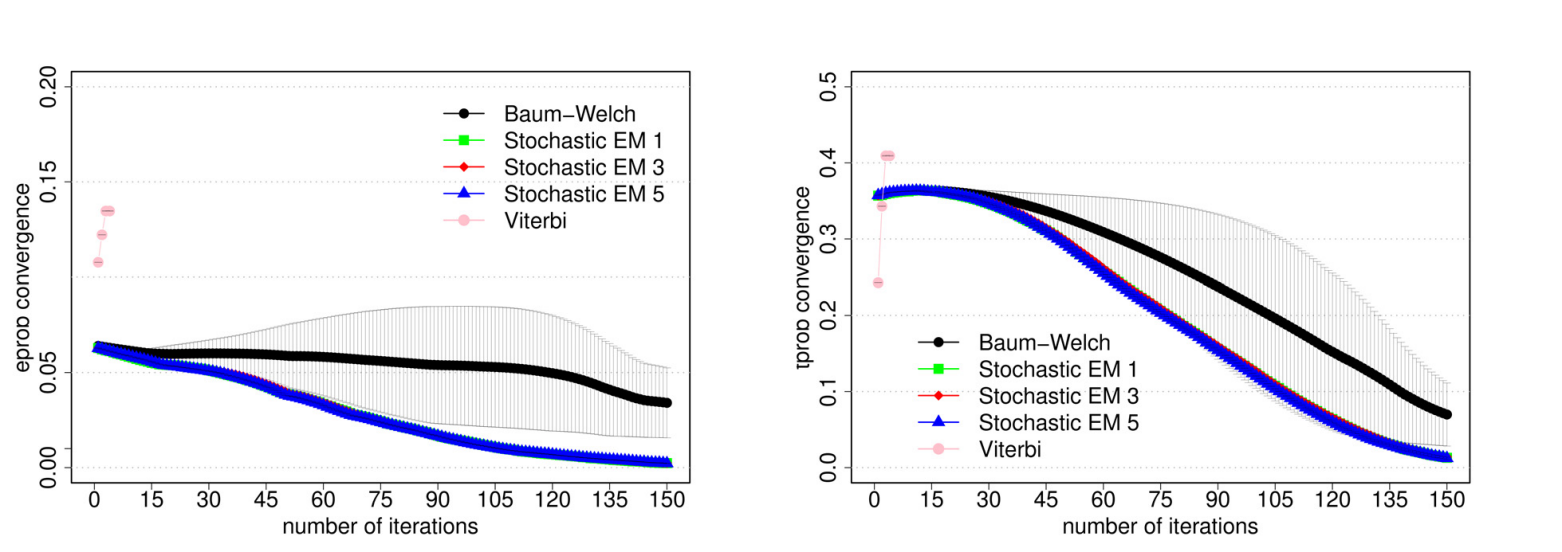
**Parameter convergence for the dishonest casino**. Average differences of the trained and known parameter values as function of the number of iterations for each training algorithm. For a given number of iterations, we first calculate the average value of the absolute differences between the trained and known value of each emission parameter (left figure) or transition parameter (right figure) and then take the average over the three experiments from the three-fold cross-evaluation. The error bars correspond to the standard deviation from the three cross-evaluation experiments. The algorithms have the same meaning as in Figure 2. Please refer to the text for more information.

The data set for this model consists of 300 sequences of 5000 bp length each. The results of the training experiments are shown in Figures 2 and 3.

#### Example 2: The extended dishonest casino

**Figure 4:**
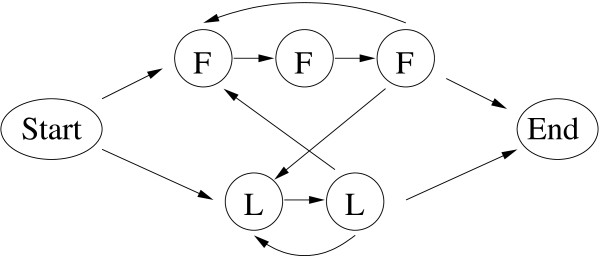
**HMM of the extended dishonest casino**. Symbolic representation of the HMM of the extended dishonest casino. States are shown as circles, transitions are shown as directed arrows. Please refer to the text for more details.

In order to investigate a HMM with a more complicated regular grammar, we extended the above example of the dishonest casino so it can now use the loaded dice (state L) only in multiples of two and the fair dice (state F) only in multiples of three, see Figure 4.

This extended HMM has seven states, the silent Start and End states, two F states and three L states, 11 transition probabilities and 30 emission probabilities. Parameterizing the HMM's probabilities yields two independent transition probabilities and 10 independent emission probabilities to be trained, i.e. 12 parameter values. In order to avoid premature termination of parameter training, we use pseudo-counts of 1 for every parameter to be trained.

**Figure 5:**
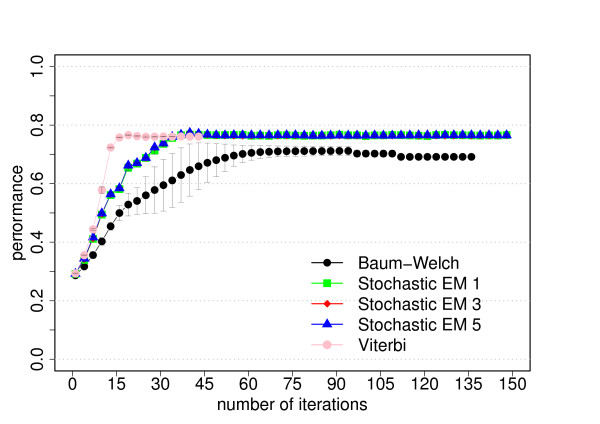
**Performance for the extended dishonest casino**. The average performance as function of the number of iterations for each training algorithm. The performance is defined as the product of the sensitivity and specificity and the average is the average of three cross-evaluation experiments. For stochastic EM training, a fixed number of state paths were sampled for each training sequence in each iteration (stochastic EM 1: one sampled state path, stochastic EM 3: three sampled state paths, stochastic EM 5: five sampled state paths). The error bars correspond to the standard deviation of the performance from the three cross-evaluation experiments. Please refer to the text for more information.

**Figure 6:**
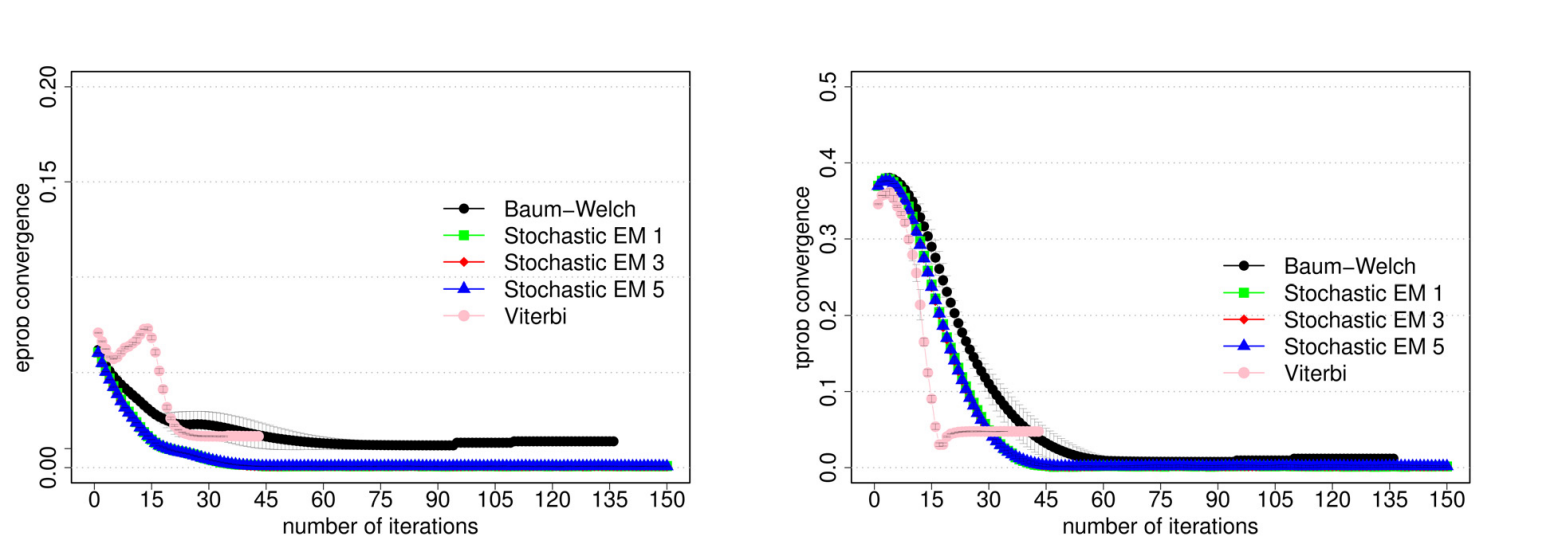
**Parameter convergence for the extended dishonest casino**. Average differences of the trained and known parameter values as function of the number of iterations for each training algorithm. For a given number of iterations, we first calculate the average value of the absolute differences between the trained and known value of each emission parameter (left figure) or transition parameter (right figure) and then take the average over the three cross-evaluation experiments. The error bars correspond to the standard deviation from the three cross-evaluation experiments. The algorithms have the same meaning as in Figure 5. Please refer to the text for more information.

The data set for this model consists of 300 sequences of 5000 bp length each. The results for this extended model are shown in Figures 5 and 6.

#### Example 3: The CpG island model

**Figure 7:**
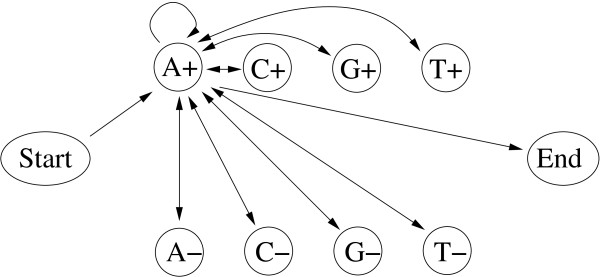
**CpG island HMM**. Symbolic representation of the CpG island HMM. States are shown as circles, transitions are shown as directed arrows. Every non-silent state can be reached from the *Start *state and has a transition to the *End *state. In addition, every non-silent state is connected in both directions to all non-silent states. For clarity, we here only show the transitions from the perspective of the A^+ ^state. Please refer to the text for more details.

In order to study the features for the different training algorithms for a bioinformatics application, we also investigate an HMM that can be used to detect CpG islands in sequences of genomic DNA [13], see Figure 7. The model consists of 10 states, the silent Start and End states, four non-silent states to model regions inside CpG islands (states A^+^, C^+^, G^+ ^and T^+^) and four non-silent states to model regions outside CpG islands (states A^−^, C^−^, G^− ^and T^−^). The emission probabilities for each of the eight non-silent states is a delta-function so that any particular state (say A^+ ^or A^−^) has an emission probability of 1 for reading the corresponding DNA nucleotide (in this case A) and a probability of zero for all other nucleotides, i.e. *e*_*X *_+ (*Y*) = *e*_*X*− _(*Y*) = *δ*_*X*,*Y *_for *X*, *Y *∈ {A, C, G, T}. This implies that none of the emission probabilities of this model thus requires training. With a total of 80 transition probabilities the model is, however, highly connected as any non-silent state is connected in both directions to any other non-silent state. Parameterizing these transition probabilities results in 33 parameters, 32 of which were determined in training (the transition probability to go to the End state was fixed). In order to avoid premature termination of parameter training, we use pseudo-counts of 1 for every parameter to be trained.

**Figure 8:**
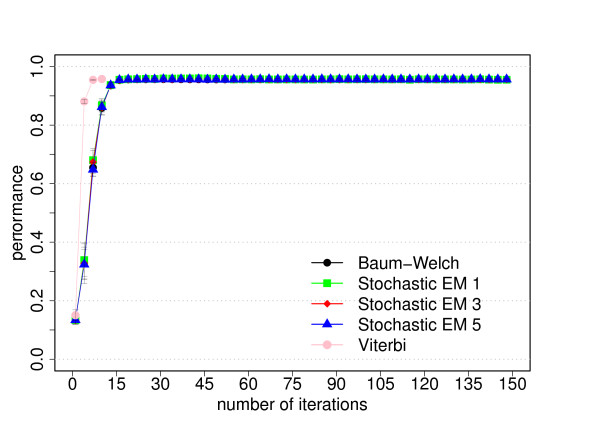
**Performance for the CpG island model**. The average performance as function of the number of iterations for each training algorithm. The performance is defined as the product of the sensitivity and specificity and the average is the average of three cross-evaluation experiments. For stochastic EM training, a fixed number of state paths were sampled for each training sequence in each iteration (stochastic EM 1: one sampled state path, stochastic EM 3: three sampled state paths, stochastic EM 5: five sampled state paths). The error bars correspond to the standard deviation of the performance from the three cross-evaluation experiments. Please refer to the text for more information.

**Figure 9:**
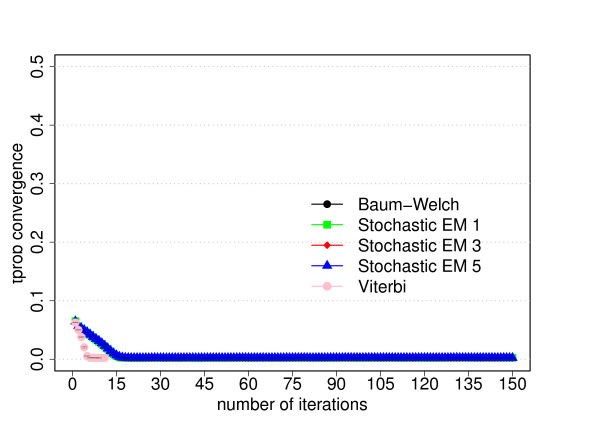
**Parameter convergence for the CpG island model**. Average differences of the trained and known parameter values as function of the number of iterations for each training algorithm. For a given number of iterations, we first calculate the average value of the absolute differences between the trained and known value of each transition parameter (this model does not have any emission parameters that require training) and then take the average over the three cross-evaluation experiments. The error bars correspond to the standard deviation from the three cross-evaluation experiments. The algorithms have the same meaning as in Figure 8. Please refer to the text for more information.

The data set for this model consists of 180 sequences of 5000 bp length each. Figures 8 and 9 show the resulting performance.

##### Prediction accuracy and parameter convergence

Our primary goal is to investigate how the prediction accuracy of the different training algorithms varies as function of the number of iterations. The prediction accuracy or performance is defined as the product of the sensitivity and specificity. Figures 2, 5 and 8 show the prediction accuracy as function of the number of iterations for all three training methods for the respective model.

Another important goal of parameter training is to recover the original parameter values of the corresponding model. We therefore also investigate how well the trained parameter values converge to the original parameter values, see Figures 3, 6 and 9 show the average differences between the trained and known parameter values as function of the number of iterations for each training algorithm and the respective model. Every data point is calculated by first determining the average value of the absolute differences between the trained and known value of each emission parameter (left figures) or transition parameter (right figures) and then taking the average over the three experiments from the three-fold cross-evaluation.

For the dishonest casino and the extended dishonest casino, stochastic EM training performs best, both in terms of performance and parameter convergence. It is interesting to note that the results for sampling one, three or five state paths per training sequence and per iteration are essentially the same within error bars. For these two models, Viterbi training converges fastest, i.e. the Viterbi paths remain the same from one iteration to the next, but the point of convergence is sub-optimal in terms of performance and in particular in terms of parameter convergence. Baum-Welch training does better than Viterbi training for these two models, but not as well as stochastic BM training as it requires more iterations to reach a lower prediction accuracy and worse parameter convergence and as it exhibits the largest variation with respect to the three cross-evaluation experiments. The latter is due to many high-scoring, sub-optimal state paths. For the CpG island model, all training algorithms do almost equally well, with Viterbi training converging fastest. Table 2 summarizes the CPU time per iteration for the different training algorithms and models. For all three models, stochastic EM training is faster than Baum-Welch training for one, three or five sampled state paths per training sequence. Viterbi training is even a bit more time efficient than stochastic EM training when sampling one state path per training sequence.

**Table 2:** CPU time use for different models

CPU time (sec) per iteration	dishonest	extended dishonest	CpG island
	Casino	Casino	Model
Baum-Welch training	8.85	5.94	22.22
stochastic EM training *K *= 1	5.12	3.42	5.42
stochastic EM training *K *= 3	6.02	4.42	10.30
stochastic EM training *K *= 5	7.06	5.38	14.84
Viterbi training	4.42	2.84	5.00

Overview of the CPU time usage in seconds per iteration for Viterbi training, Baum-Welch training and stochastic EM training for the three different models. For each model, we implemented each of the three training methods using the linear-memory algorithms for Baum-Welch training, Viterbi training and stochastic EM training. The number of state paths that are sampled for each iteration and each training sequence in stochastic EM training is denoted *K*.

Based on the results from these three small example models, we would thus recommend using stochastic EM training for parameter training.

## Conclusion and discussion

A wide range of bioinformatics applications are based on hidden Markov models. Having computationally efficient algorithms for training the free parameters of these models is key to optimizing the performance of these models and to adapting the models to new data sets, e.g. biological data sets from a different organism.

We here introduce two new algorithms which render the memory requirements for Viterbi training and stochastic EM training independent of the sequence length. This is achieved by replacing the usual bi-directional two-step procedure (which involves first calculating the Viterbi matrix and then retrieving the Viterbi path (in case of Viterbi training) or first calculating the forward matrix and the backward matrix before estimating counts (in case of Baum-Welch training)) by a one-step procedure which scans each training sequence only in a one-directional way. For an HMM with *M *states and a connectivity of *T*_*max*_, a training sequence of length *L *and one iteration, our new algorithm reduces the memory requirement of Viterbi training from O(*ML*) to O(*M *) while keeping the time requirement of O(*MT*_*max*_*L*) unchanged, see Table 1 for details. For stochastic EM training where *K *is the number of state paths sampled for every training sequence in every iteration, the memory requirements are (as, typically, *L *≫ *K *+ 1 ≥ *K *+ *T*_*max*_/*M *) reduced from O(*ML*) to O(*MK + T*_*max*_) while the time requirement per iteration changes from O(*T*_*max*_*L*(*M + K*)) to O(*T*_*max*_*LMK*) depending on the user-chosen value of *K*. An added advantage of our two new algorithms is they are easier to implement than the corresponding default algorithms for Viterbi training and stochastic EM training. In addition to introducing the two new algorithms for Viterbi training and stochastic EM training, we also examine their practical merits for three small example models by comparing them to the linear-memory algorithm for Baum-Welch training which was introduced earlier. Based on our results from these three (non-representative) models, we would recommend using stochastic EM training for parameter training.

We have implemented the new algorithms for Viterbi training and stochastic EM training as well as the linear-memory algorithm for Baum-Welch training into our HMM-compiler HMMCONVERTER[37] which can be used to set up a variety of HMM-based applications and which is freely available under the GNU General Public License version 3 (GPLv3). Please see http://people.cs.ubc.ca/~irmtraud/training for more information and the source code.

We hope that the new parameter training algorithms introduced here will make parameter training for HMM-based applications easier, in particular those in bioinformatics.

## Competing interests

The authors declare that they have no competing interests.

## Authors' contributions

TYL and IMM devised the new algorithms, TYL implemented them, TYL and IMM conducted the experiments, evaluated the experiments and wrote the manuscript. All authors read and approved the final manuscript.
